# Validation of ultrasound bioimaging to predict worm burden and treatment efficacy in preclinical filariasis drug screening models

**DOI:** 10.1038/s41598-018-24294-2

**Published:** 2018-04-12

**Authors:** Amy E. Marriott, Hanna Sjoberg, Hayley Tyrer, Joanne Gamble, Emma Murphy, John Archer, Andrew Steven, Mark J. Taylor, Joseph D. Turner

**Affiliations:** Research Centre for Drugs and Diagnostics, Department of Parasitology, Liverpool School of Tropical Medicine, Pembroke Place, Liverpool, L3 5QA UK

## Abstract

Filariasis is a global health problem targeted for elimination. Curative drugs (macrofilaricides) are required to accelerate elimination. Candidate macrofilaricides require testing in preclinical models of filariasis. The incidence of infection failures and high intra-group variation means that large group sizes are required for drug testing. Further, a lack of accurate, quantitative adult biomarkers results in protracted timeframes or multiple groups for endpoint analyses. Here we evaluate intra-vital ultrasonography (USG) to identify *B. malayi* in the peritonea of gerbils and CB.17 SCID mice and assess prognostic value in determining drug efficacy. USG operators, blinded to infection status, could detect intra-peritoneal filarial dance sign (ipFDS) with 100% specificity and sensitivity, when >5 *B. malayi* worms were present in SCID mice. USG ipFDS was predictive of macrofilaricidal activity in randomized, blinded studies comparing flubendazole, albendazole and vehicle-treated SCID mice. Semi-quantification of ipFDS could predict worm burden >10 with 87–100% accuracy in SCID mice or gerbils. We estimate that pre-assessment of worm burden by USG could reduce intra-group variation, obviate the need for surgical implantations in gerbils, and reduce total SCID mouse use by 40%. Thus, implementation of USG may reduce animal use, refine endpoints and negate invasive techniques for assessing anti-filarial drug efficacy.

## Introduction

The filarial neglected tropical diseases (NTDs), lymphatic filariasis (LF) and onchocerciasis are prioritized for elimination by the World Health Organization by 2030, in line with The United Nations Sustainable Development Goals^1,2^. LF, caused by *Wuchereria bancrofti, Brugia malayi* and *B. timori* affects an estimated 120 million people^3^ with another billion people at risk of infection^4^. Clinically, the disease presents as elephantiasis and hydrocele in approximately 40 million LF patients, and is a major cause of global disability^2^. Onchocerciasis is caused by the related filaria, *Onchocerca volvulus* and infects an estimated 37 million individuals, mainly in Sub-Saharan Africa. In its most severe presentation, ocular onchocerciasis (river blindness), which affects 0.8 million people, is a major cause of preventable blindness^1^.

Current strategies to eliminate LF and onchocerciasis are centered around The World Health Organization’s (WHO) Expanded Special Project for Elimination of NTDs, in which repeated doses of preventable chemotherapies (PCT) are administered annually to endemic populations through mass drug administration (MDA) campaigns^5^. These PCT drugs, namely ivermectin, albendazole and diethylcarbamazine (DEC), target the transmissible larval stage of the parasites the microfilaria (mf). Although effective PCT treatments temporarily induce sterilization of adult worms, they do not (in current MDA recommended regimens) mediate significant macrofilaricidal (curative) activity. With a reproductive life-span of 5 years (LF)^6^ or 10 years (onchocerciasis)^7^. MDA has to be delivered with high population coverage for at least this period of time to deliver transmission break points. Issues of adherence to treatment, problems of sustained drug delivery and development of drug resistance have culminated in the lack of expected elimination outcomes in certain endemic foci^8–10^.

A recognized solution to this problem is the development of new drugs for filariasis which can mediate curative efficacy with following a short-course, test-and-treat strategy. New drug candidates (either repurposed registered drugs or new chemical entities) require rigorous assessment of efficacy in preclinical models of filariasis. Current standard screening models use either surrogate species (the cotton rat filaria *Litomosoides sigmodontis* and the dog filaria, *B. pahangi*) or a human sub-periodic strain of *B. malayi*. All three species of lymphatic filariae can establish long-term, fecund infections (≥8 months) in mongolian gerbils following inoculation with infectious stage larvae. *L. sigmodontis* adults infect the pleural cavity via migration through the lymphatics, whilst *Brugia spp* establish in either the peritoneal cavity or the lymphatics, depending on route of infection. For *L. sigmodontis*, shorter term murine infections can be established (for 2–3 months) in BALB/c inbred mice. Also for *Brugia spp*., use of immunodeficient mice (such as CB.17 SCID) can allow for long-term adult filarial survival in the peritoneum, similar to duration of chronic infections in gerbils, (≥8 months)^11–13^.

In all models, large variation is evident in the adult parasite success rate from a unit inoculate per animal. Variation is comprised of both negative binomial distribution (skewness) and a low occurrence of non-parasitized animals (where inoculates have either failed to establish adult infections or where adult infections have only transiently established). Currently, accurate quantitative biomarkers of present adult infections are lacking and thus rodents cannot be assessed for infection status or parasitic load prior to enrolment in drug screening. Similarly, due to the lack of accurate markers of present adult infection, drug efficacy can only be evaluated at the end-point of the experiment, through dissection. To compensate, current screening protocols have long washout durations to maximize chances of capturing the ‘true’ efficacy of ‘slow-acting’ macrofilaricides, with concomitant risk of reduced survival of remaining adult filariae due to host attrition (independent of drug effect) and/or risk of decline in animal welfare. These drawbacks result in the requirement for large experimental group sizes, increased costs of maintenance, high demand on complex parasite production and protracted iterative cycles, in order to provide decision-making efficacy outputs (e.g. to support pharmaceutical lead-optimisation programmes).

Clinical ultrasonography (USG) has been used in tropical medicine to diagnose and assess therapeutic success in numerous diseases^14^. USG is particularly amenable in the detection of macroparasitic tissue infections due to the large size of the pathogens, their distinctive motility and/or the frequent formation of a cystic space in the tissues they inhabit e.g. echinococcosis, cysticercosis and filariasis. In LF, Amaral *et al* first described the random thrashing movements of adult *W. bancrofti* as the filarial dance sign (FDS) in dilated lymphatic vessels during scrotal USG^15^. *W. bancrofti* USG detection of FDS has been used to diagnose suspected and unsuspected cases of scrotal filariasis^16,17^, and has been an important tool for the evaluation of macrofilaricide and anti-morbidity drugs^18–22^. The reproducible success of USG in detecting FDS in bancroftian filariasis has only been reported in the scrotal region in microfilariaemic males, with more inconsistent detection in the lymphatics of other anatomical locations of microfilaraemic female patients^23^. Similarly, *B.malayi*, which are smaller than *W. bancrofti* worms (4 cm *versus* 10 cm) and do not form hydrocoele pathology, have failed to be consistently visualized in microfilariaemic patients^24^.

*O. volvulus* adult motility within subcutaneous nodules have also been successfully imaged using USG. Nodules have been subjected to USG as a method of diagnosis and assessing drug treatment efficacy by determining changes in nodular structure, as well as imaging the worm motility within cystic spaces inside the nodules^25–32^.

In a pilot study utilizing rodents infected with *L. sigmodontis* or *B. malayi* and pre-determined as positive for circulating microfilaraemia, the detection of FDS by USG has been demonstrable with more reliable detection in the thoracic cavity *versus* the lymphatic system^33^. Here, we have fully assessed the sensitivity, specificity and prognostic value of USG to detect FDS of extravascular *B. malayi* adult filariae contained within the peritoneal cavity of SCID mouse and gerbil preclinical drug screening systems. We demonstrate that USG is highly sensitive and specific in the detection of FDS using ‘operator-blinded’ studies and can be successfully applied to estimate level of adult worm burden and macrofilaricidal efficacy in drug screening experiments.

## Materials and Methods

### Animals

Male CB.17 Severe Combined ImmunoDeficient (SCID) mice were purchased from Charles River UK. *Meriones unguiculatus* (Mongolian gerbils; jirds) breeding pairs were purchased from Charles River, Europe. Breeding and experimental stocks were maintained under specific pathogen-free (SPF) conditions at the biomedical services unit (BSU), University of Liverpool, Liverpool, UK. Male SCID mice were 6–10 weeks old and weighed 22–26 g at start of experiments. Male gerbils were 4–6 months old and weighed 80–100 g at start of experiments. All experiments were approved by the ethical committees of the University of Liverpool and Liverpool School of Tropical Medicine (LSTM) and conducted under Home Office Animals (Scientific Procedures) Act 1986 (UK) requirements. Animal experiments were undertaken in accordance with NC3Rs ARRIVE guidelines.

### Brugia malayi parasite production

The life cycle of *Brugia malayi (*Bm) was maintained in mosquitoes and Mongolian gerbils. For *Bm* larvae (*Bm*L3) generation, microfilariae (mf) were collected from infected gerbils via catheterisation. For this, Mongolian gerbils were anaesthetized with isoflurane and subjected to peritoneal washes with RPMI 1640 media (ThermoFisher Scientific) to harvest mf. Mf were then purified using PD10 column size exclusion chromatography (Amersham), enumerated by microscopy and mixed with human blood to a final concentration of 15–20,000 mf/ml. Mf were then fed to female *Aedes aegypti* mosquitoes through an artificial membrane feeder (Hemotek). Blood fed mosquitoes were reared for 14 days with daily sugar-water feeding to allow development to *Bm*L3 stage. At day 14, *Bm*L3 were collected from infected mosquitoes by crushing and concentration using a Baermann’s apparatus and RPMI media.

### Brugia malayi Experimental Infections

For *Bm*L3 infection, male Mongolian gerbils, aged 4–6 months, were injected via the intraperitoneal route, with with either 50 or 400 highly motile *Bm*L3. Male CB.17 SCID mice aged 6–10 weeks, were injected with 100 *Bm*L3. Animals were left for between 12 and 25 weeks post-infection to allow infections to proceed to the chronic adult stage.

### Surgical implantation of Adult Brugia malayi parasites

*Bm* adults were collected from infected donor CB.17 SCID mice via peritoneal lavage post-mortem. Parasites were then separated into male and female, washed with pre-heated phosphate buffered saline (PBS, Merck) and collected into the following groups: 4x5 males, 4x5 females, 4x2 males, 4x2 females, 4x1 male, 4x1 female, for implantation (n = 4/group, total n = 28). Male SCID mice were then placed under surgical anesthesia using isofluorane and received a subcutaneous injection of buprenorphine prior to implantation of the above parasite groups into the peritoneal cavity. Implantation was achieved by making a small incision into the skin and abdominal cavity in the upper right quadrant and inserting parasites into the lower abdominal quadrant using a glass pipette to ensure all parasites were maintained in the cavity. The incisions were then re-sutured after implant and animals were re-housed as before and monitored closely. The number of parasites inserted into each mouse was blinded to the investigator and coded by ear markings for parasite recovery analysis at the experimental end point.

### Preclinical Ultrasonography

To initially optimise the USG technique, a cohort of 5 CB.17 SCID mice were surgically implanted with 13 adult *Bm* parasites and imaged before and after the addition of sterile RPMI media to establish a ipFDS signal. Similarly, 8 gerbils infected with varying numbers of *Bm*L3 were imaged before and after 1 and 3 ml of RPMI to optimize the imaging protocol. To further assess the accuracy of USG in detecting *Bm* parasites and to determine limits of sensitivity in CB.17 SCID mice, USG was performed blinded one week post-surgery. For this, mice and gerbils were anaesthetized with gas isofluorane prior to receiving a 1 ml or 3 ml, respectively, intra-peritoneal injection of sterile, pre-heated (37 °C) RPMI media (Merck, UK). The abdominal cavity was then gently massaged to distribute the media and dislodge parasites into fluid pockets to enable easier detection with USG. The abdominal region was then shaved and imaged by USG (Sonosite® MTurbo® 8.5 Mz linear probe, ‘small parts’ preset) for 10–15 minutes with thorough investigation of all quadrants for random thrashing movements (ip filarial dance sign; ipFDS) to confirm the presence of parasitic worms. Parasite load and location was semi-quantified using a grid scoring method depending on signal strength and number of locations in which parasite masses were detected. Animals were scored as ipFDS- (no FDS detection), ipFDS+ (a single location with a weak signal), ipFDS++ (a weak signal at >1 locations) or ipFDS+++ (a strong signal at ≥1 locations). For the initial validation of ipFDS, the abdomen was firstly imaged using ‘M-mode’ of USG, the time motion display, to accurately confirm parasite presence and location due to the recording of very rapid movements exhibited by *B. malayi* FDS, which are not observed with artifacts of respiration, intestinal peristalsis or blood flow. For further validation, the ‘Pulse Wave’ modality was applied to depict velocity and flow direction both as a waveform. For this, FDS could be confirmed due the random movements and velocity, as opposed to erythrocytes, which instead exhibit a constant flow and velocity. For the Colour Flow Doppler, the same parameters were examined as a colour map superimposed onto the 2D imaged, whereby flow moving away from the probe can by determined in one colour and flow away determined by another. The random FDS of *B. malayi* allows for colours to change more rapidly during video, and more flow observed moving away from the probe.

### Drug treatments

Individual mice (unit of replication; n = 5/group) were randomized into treatment groups by ear notch ID (001, vehicle, 002 ABZ, 003 FBZ etc.) Mice were dosed with either a known potent macrofilaricidal parenteral regimen of flubendazole (FBZ); 10 mg/kg daily s.c. × 5 days, a related BZ drug regimen of albendazole (ABZ) not expected to confer macrofilaricidal activity, at 5 mg/kg twice daily per oral for 7 days or with vehicle matching ABZ for 7 days. USG was carried out by an investigator blinded to treatment to detect presence or absence of ipFDS as a prognostic marker of macrofilaricidal outcome of the drug screen at +6 weeks.

### Endpoint Parasitological assessments

At indicated intervals post-USG imaging, animals were humanely culled before adult *B. malayi* were recovered by extensive peritoneal washes with RPMI medium. Parasite numbers were sexed and counted by microscopy. Motility scoring was based on a system whereby 3 = highly vigorous movements. 2 = slow movements, 1 = twitching, 0 = immotile. Metabolic activity of adult *B. malayi* recovered at necropsy were determined by washing in PBS and individually placing in a solution of MTT (3-(4,5-Dimethylthiazol-2-yl)-2,5-Diphenyltetrazolium Bromide) reagent (Merck) in PBS (final concentration 0.5 mg/ml). Worms were incubated for 2 hours at 37 °C with 5% CO_2_. After washing in PBS, adult worms were incubated in 100% DMSO for 1 hour at 37 °C with 5% CO_2_ to dissolve and release the blue formazan product. The samples were read at OD 490 nm on a 96-well plate reader (Varioskan, Bio-Rad).

### Statistics

Raw or log transformed continuous variables were tested for normal distribution using D’Agostino & Pearson omnibus normality tests. Variables that passed normality tests were analyzed by 1 way ANOVA with Holm-Sidak’s multiple comparisons tests. Variables significantly different from a normal distribution were analyzed by Kruskal-Wallis with Dunn’s multiple comparisons tests. Differences in frequency of categorical variables were assessed by Chi-Square analysis. Significance was defined at alpha <0.05 and analyzed using GraphPad Prism v6.0h. Power analysis was undertaken using sample means and standard deviations of untreated/vehicle control gerbil or SCID mouse worm burdens combined from 2–3 independent infection or implantation experiments. With the assumption of proportional variation, sample size was calculated for drug efficacy effect sizes of 70% or 90% with a statistical power (1-ß) of >75 < 90% with alpha set at 0.05 using a two-sample T test (Russ Lenth PiFace Applet: https://homepage.divms.uiowa.edu/~rlenth/Power/index.html).

## Results

### Optimisation and characterisation of intra-peritoneal FDS detection

**Figure 1:**
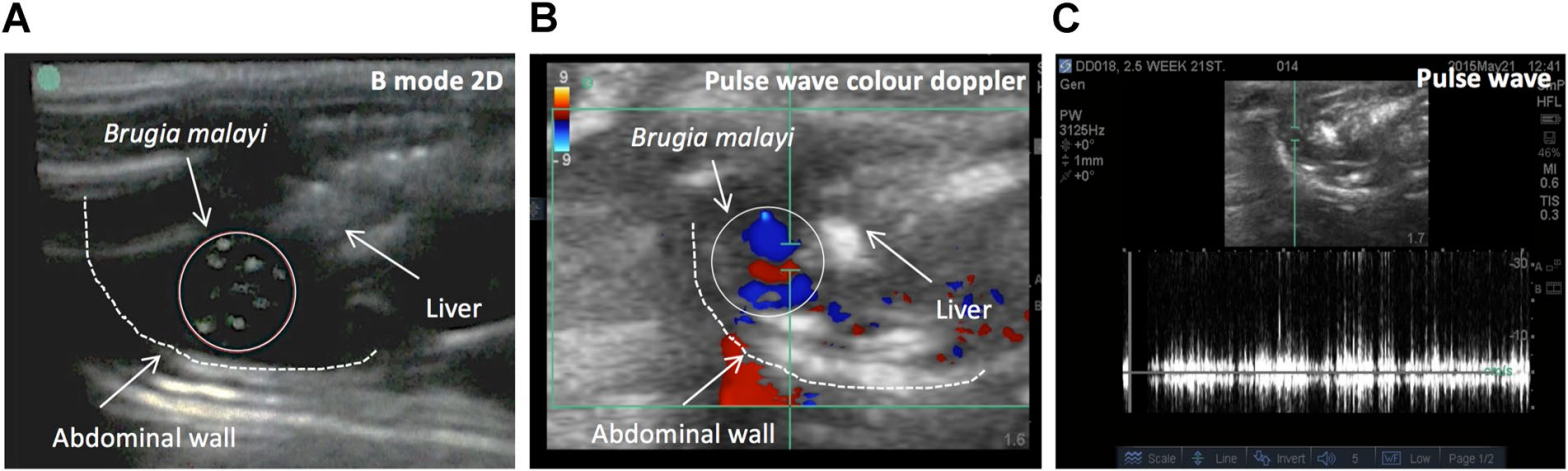
USG identification of rodent intraperitoneal filarial dance sign. Presence of *B. malayi* worm clusters with rapid motility detected in B mode within cystic spaces between viscera and abdominal wall of SCID mice (**A**). Irregular *B. malayi* motility (filarial dance sign) captured by pulse wave color doppler (**B**) and pulse wave (**C**). Movements away from the transducer probe in blue, those towards the transducer in red (**B**).

Initial experimentation was undertaken to optimise the detection of adult *B. malayi* FDS within the peritoneum. *B. malayi* immature adult stages were aseptically isolated from SCID mice +6 weeks following infection with 100 L3 ip. Six weeks after *B. malayi* immature adult stages had been surgically implanted into recipient SCID mice (adult filariae = +12 weeks old), mice were anaesthetised, orientated in a supine position, abdominal hair removed by shaving and abdomen imaged with a Sonosite Mturbo portable USG with 8.5 Mz linear probe. After a maximum of 15 minutes imaging, intra-peritoneal (ip) FDS detection was verified in 2/5 animals (Table 1). Mice were then injected with 1 ml of pre-warmed, sterile RPMI medium ip, and the peritoneum was gently massaged before re-imaging for a further 15 minutes. After this intervention, 5/5 animals had detectable ipFDS signal, most frequently observed in the upper right or upper left abdominal quadrants in cystic spaces between the abdominal wall and viscera using USG M-mode. For further confirmation of FDS, Doppler modalities were applied, whereby the randomised movements and velocity of FDS were confirmed through a ‘filled in’ Doppler signal (Fig. 1C), whereas laminar flow, exhibited by normal blood flow and respiration patterns display only a thick white edge, with black within. The Colour Doppler mode also further validated FDS signal with the blue colour exhibiting the random movement and direction of parasites moving quickly away from and towards the probe, against the red colour which signifies normal blood flow, peristalsis and respiration, with these moving only towards the probe in one direction (Table 1A, Fig. 1A–C and S video 1–3). Similarly, four Mongolian gerbils, that had chronic adult infections 3 months post-infection with 400 *Bm*L3, received a 1ml injection before imaging, in which no animals scored positively for ipFDS. When a further 2 ml (total of 3 ml) media was injected into the peritoneal cavity, 4/4 gerbils had detectable ipFDS (Table 1).

**Table 1:** Optimisation of intra-peritoneal FDS detection by ultrasound in anaesthetised mice or gerbils.

ID	ipFDS signal	ipFDS signal (+1 ml medium ip)	ipFDS signal (+3 ml medium ip)	n adult *Bm* recovered
SCID 1	−	+	nd	6
SCID 2	+	+	nd	6
SCID 3	+	+	nd	7
SCID 4	−	+	nd	7
SCID 5	−	+	nd	4
Gerbil 1	−	−	+	2
Gerbil 2	−	−	+	2
Gerbil 3	−	−	+	12
Gerbil 4	−	−	+	67

### Sensitivity and specificity of *B. malayi* adult parasite loads by USG in operator-blinded studies

To assess sensitivity and specificity of USG in the detection of ipFDS, including low level worm burdens, CB.17 SCID mice were surgically implanted ip with either 10 female and 5 male (n = 16), five female, five male, two female, two male, one female or one male *B. malayi* (all n = 5/group). All *B. malayi* used were +12–13 weeks old sourced from CB.17 SCID donors infected with *Bm*L3. A further five SCID mice were submitted to peritoneal surgery and sham implantations. Between one and five weeks post-surgery, animals were imaged by USG for +15 minutes under anaesthesia following introduction of 1 ml pre-warmed medium and peritoneal massage. Mice were imaged in random order, by one of two operators who were blinded to group. The following day post-USG imaging, mice were necropsied and numbers of motile male and female adult *B. malayi* enumerated. Table 2 details the sensitivity and specificity of USG in detecting adult *B. malayi* parasites. In Table 2, numbers listed in ‘*Bm* females’ or ‘*Bm* males’ columns denotes numbers of female or male parasites recovered from individual mice and commas separate parasite yields collected from individual mice. In total, at necropsy, 42/46 mice had retained one or more motile *B. malayi* adults post-implantation. Therefore, combined with sham implants, a total of 9 mice were confirmed to lack motile *B. malayi*. The USG operators were able to accurately predict the absence of motile *B. malayi* in the peritoneum of all 9 infection negative mice (no false positives; 100% specificity). The operators predicted the presence of motile *B. malayi* in 36/42 mice (6 false negatives; 85.7% sensitivity). When examining the false negative rate according to final worm burden at necropsy, the operators were able to predict with 100% sensitivity the presence of >5 adult, motile *B. malayi*. This sensitivity dropped to 81% when ≤5 adult parasites were present in the peritoneum. Comparing the ability of USG to detect low level (≤5) female *versus* male implants, the level of sensitivity was similar (77%, female implants *versus* 82%, male implants). USG was reproducibly able to detect single motile female (7/9) or male (5/5) *B. malayi* within the peritoneal cavity of mice.

**Table 2:** Sensitivity and specificity of USG in determining adult motile *B. malayi*.

total adult *Bm* recovered (n)	*Bm* females	*Bm* males	Mice n	Mice ipFDS+	Mice ipFDS−
12	9,9	3,3	2	2	0
10	9	1	1	1	0
9	7,7	2,2	2	2	0
8	8,6,5	0,2,3	3	3	0
6	4,4,3	2,2,3	3	3	0
5	3	2	1	1	0
4	2	2	1	1	0
2	1	1	1	1	0
5	5		1	1	0
4	4,4		2	2	0
3	3		1	0	1
2	2,2,2,2		4	3	1
1	1,1,1,1,1,1,1,1,1		9	7	2
5		5,5	2	0	2
4		0	0	0	0
3		3,3	2	2	0
2		2,2	2	2	0
1		1,1,1,1,1	5	5	0
0 (including sham)	0	0	9	0	9
**total** ***Bm*** **+**			**42**	**36**	**6**
**total** ***Bm−***			**9**	**0**	**9**
**Total**			**51**	**36**	**15**
**sensitivity**				**85.7%**	
sensitivity >5 adult *Bm*				100%	
sensitivity ≤5 adult *Bm*				80.6%	
sensitivity ≤5 female *Bm*				76.5%	
sensitivity ≤5 male *Bm*				81.8%	
**specificity**					**100%**

### Validation of USG to predict macrofilaricidal activity in preclinical drug screening

We utilised a known macrofilaricidal regimen of flubendazole compared with a known non-macrofilaricidal anthelmintic regimen of oral albendazole to test whether USG could accurately predict macrofilaricidal activity in live animals during drug screening experiments^11,34^. In both experiments, individual USG operators were blinded to drug group and imaging assessments of mice occurred in random order. In experiment A, mice were implanted with 10 female and 5 male worms and the USG signal was assessed +6 weeks after start of treatment. Necropsies were performed immediately after USG to determine parasite worm burden. Metabolic activity and motility assessments of surviving females worms were also undertaken (Table 3). Following de-blinding, ipFDS signal was determined in 8/8 vehicle control treated mice, 8/8 ABZ treated mice and 3/9 FBZ treated mice. The frequency of ipFDS detection was significantly lower in the FBZ group compared with vehicle or ABZ (*P* = 0.0009). The USG findings were then compared with the parasitological readout of the drug screen, at termination (Table 3). In all vehicle and ABZ treated animals, motile adult *B. malayi* were recovered. The frequency of infection in FBZ treated mice was significantly reduced (4/9 mice, *P* = 0.0039). Evaluating total worm burden, in the vehicle control group, a median of 7 (range 2–12) motile adult *B. malayi* were recovered per mouse. A similar median recovery was evident in the ABZ group, whereas in the FBZ treatment group, median worm burden was significantly reduced (*P* = 0.0003) with the four mice that remained infection positive containing a single female worm. USG signal was detected in 3/4 of these mice. Further, motility assessments *ex vivo* evaluated that the majority of adult female *B. malayi* derived from vehicle and ABZ groups retained vigorous motility whilst the four *B. malayi* surviving in the FBZ group displayed a significantly reduced moribund motile phenotype, indicating a reduction in viability (*P* < 0.00001).

**Table 3:** USG ipFDS detection compared with adult *B. malayi* parasitological readouts in experimental macrofilaricide drug screens.

Drug group^+^	USG ipFDS+/total (weeks post-dosing)	Infection status (weeks post-dosing)	median *B. malayi* worm burden (range, total n)	Median Female *B. malayi* worm burden (range, total n)	Median Male *B. malayi* worm burden (range, total n)	Mean Female *B. malayi* metabolic activity (SEM, n worms assessed)	Median Female *B. malayi* motility score^ (range, total n worms assessed)
**EXPT A (implant drug screen, USG operator 1)**							
Vehicle	8/8 (6 weeks)	8/8 (6 weeks)	7 (2–12, 57)	4.5 (1–9, 40)	2 (0–3, 17)	0.40, 39 (0.002)	3 (1–3, 39)
ABZ	8/8 (6 weeks)	8/8 (6 weeks)	7 (2–12, 53)	5 (0–9, 39)	2 (0–3, 14)	0.48, 39 (0.07)	3 (1–3, 39)
FBZ	3/9* (6 weeks)	4/9^|^ (6 weeks)	0^†^ (0–3, 4)	0 (0–3, 4)	0 (0–0)	nd	1^œ^ (1–1, 4)
**EXPT B (infection drug screen, USG operator 2)**							
Vehicle	5/5 (2.5 weeks)	5/5 (6 weeks)	18 (9–23, 85)	10 (6–15, 53)	7 (3–8, 31)	0.69, 10 (0.08)	3 (2–3, 10)
ABZ	5/5 (2.5 weeks)	5/5 (6 weeks)	19 (14–21, 93)	14 (11–16, 67)	5 (3–6, 24)	0.28^∞^, 10 (0.05)	3 (2–3, 10)
FBZ	1/5^§^ (2.5 weeks)	4/5 (6 weeks)	1^#^ (0–4, 7)	1 (0–4, 7)	0 (0–0, 0)	0.01^∞^, 7 (0.003)	1^*∫*^ (1–2, 7)

^**+**^ABZ = albendazole 5mg/kg twice daily per oral x 7d, FBZ = flubendazole 10mg/kg once daily sc x 5d.

^**^**^Motility score: 3 = vigourously motile, 2 = sluggishly motile, 1 = partial twitching motility, 0 = immotile.

^*^Chi-square analysis *X*^2^ = 14.04*, df*,2 *P=*0.0009.

^|^Chi-square analysis *X*^2^ = 11.11*, df*,2 *P=*0.0039.

^**†**^Kruskal Wallis 1 way ANOVA 16.09, *P=*0.0003 (Dunn’s tests: vehicle *vs* FBZ, *P* < 0.01, ABZ *vs* FBZ, *P* < 0.01).

^***œ***^Kruskal Wallis 1 way ANOVA 18.49, *P* < 0.0001 (Dunn’s tests: vehicle *vs* FBZ, *P* < 0.0001, ABZ *vs* FBZ, *P* < 0.0001).

^**§**^Chi-square analysis *X*^2^ = 10.91, *df*,2 *P* = 0.0042.

^**#**^Kruskal Wallis 1 way ANOVA 40.13, *P* < 0.0001 (Dunn’s tests: vehicle *vs* FBZ, *P* < 0.0001, ABZ *vs* FBZ, *P* < 0.0001).

^**∞**^1 way ANOVA *F* = 23.93*, P* < 0.0001 (Holm-Sidak’s tests: vehicle *vs* ABZ, *P* < 0.001, vehicle *vs* FBZ, *P* < 0.0001, ABZ *vs* FBZ *P* < 0.05).

^***∫***^Kruskal Wallis 1 way ANOVA 20.47, *P* < 0.0001 (Dunn’s tests: vehicle *vs* FBZ, *P* < 0.001).

In experiment B, mice were infected with 100 *Bm*L3 and after adult infections had established (+7 weeks), mice were randomised and treatment commenced. Due to the success of USG to predict profound macrofilaricidal activity of FBZ immediately before end-point, in experiment B USG was performed at +2.5 weeks post-treatment, 3.5 weeks prior to end-point, in order to evaluate the prognostic potential of ipFDS signal detection in predicting macrofilaricidal drug activity. A different USG operator undertook evaluations in experiment B. Following de-blinding of treatment groups, USG undertaken at +2.5 weeks post-dosing detected ipFDS signal in 5/5 vehicle and ABZ treated mice. A significantly reduced frequency of ipFDS, in 1/5 FBZ treated mice, was detected (*P* = 0.0042). At the end-point, +6 weeks post-dosing, the frequencies of infection positive mice were similar between treatment groups (5/5, 5/5 and 4/5) for vehicle, ABZ and FBZ, respectively. However, total adult *B. malayi* worm burden was significantly reduced in FBZ treated animals compared with vehicle (median recovery 1 *versus* 18, *P* < 0.0001), whilst worm burdens in the ABZ group remained similar (median worm recovery = 19). In the FBZ group, one mouse had four female worms recovered whilst an additional three mice contained a single female *B. malayi*. Motility assessments of recovered adult female worms determined that the majority of *B. malayi* in vehicle and ABZ treatment groups retained vigorous motility whilst the surviving FBZ-treated *B. malayi* displayed significantly reduced and moribund ‘twitching’ motility (*P* < 0.0001). Metabolic activity of sampled female worms was also significantly reduced in both drug groups *versus* vehicle but to a more profound extent in the surviving FBZ-treated *B. malayi versus* ABZ-treated worms (*P* < 0.0001).

### Application of USG to semi-quantify *B. malayi* adult parasite loads *in vivo*

**Figure 2:**
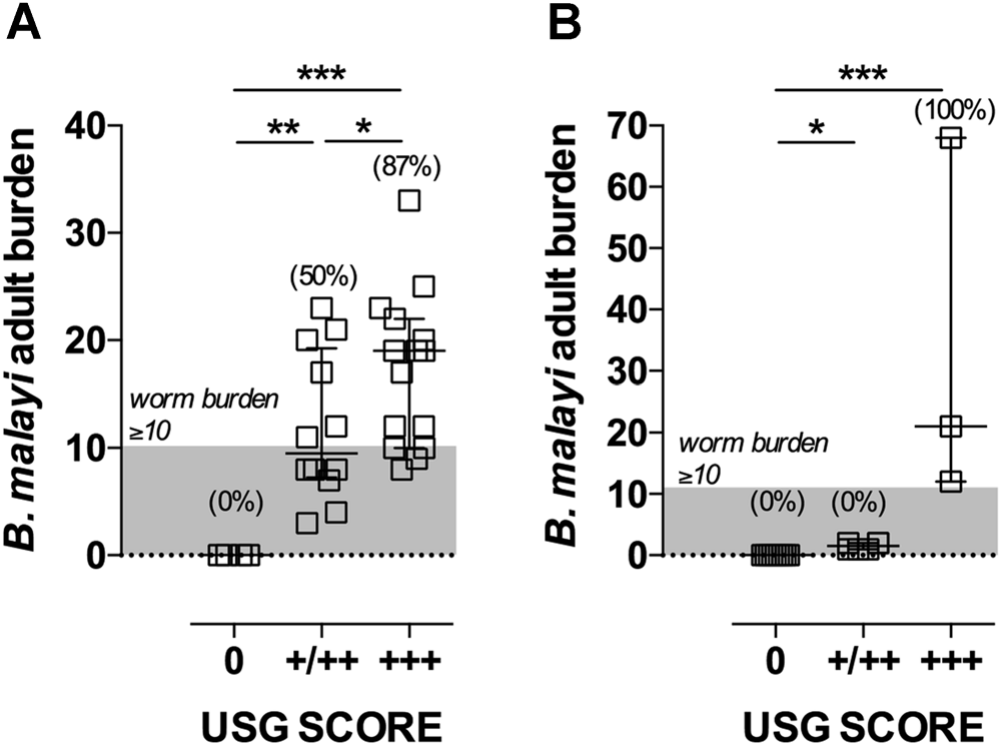
Semi-quantification of *B. malayi* worm burden by USG in SCID mouse and gerbil drug screening models. positive and negative ipFDS and ipFDS signal strength, semi-quantified in terms of number of anatomical locations and density (+/++ = low-intermediate signal, +++ = strong signal) compared with worm burdens of motile adult *B. malayi* determined at necropsy in 31 parasitised SCID mice (**A**) or 18 Mongolian gerbils (**B**). Horizontal bars represent median values and bars represent interquartile range. Percentages in parentheses are numbers of animals in each USG sub-category with a *B. malayi* worm burden ≥10 (above shaded area of graph). Significant differences were assessed by 1 way ANOVA with Holm Sidak’s multiple comparison’s test (**A**) or Kruskal Wallis with Dunn’s multiple comparison’s tests (**B**). Significant differences are indicated *P < 0.05, **P < 0.01, ***P < 0.001.

We investigated whether the qualitative signal strength of ipFDS determined by USG could be utilised to semi-quantify variation in *B. malayi* adult worm burden that arises following experimental infection with *Bm*L3. Cohorts of CB.17 SCID mice (n = 31) or gerbils (n = 14) were assessed after experimental infection and at time-points following adult parasite establishment in the peritoneum. Additionally, a group of 4 uninfected gerbils were evaluated (operator blinded to infection status). A semi-quantitative scoring system was devised based on number of discreet ipFDS signals in anatomical locations of the 4 quadrants of the peritoneum, and also apparent density of ipFDS signal, with a low-intermediate signal (+/++) inferring a low density of parasites in either a single or multiple peritoneal quadrants and a strong signal (+++) inferring a dense mass of parasites in either a single or multiple quadrants. At 40 weeks following infection, 4/31 mice had no detectable ipFDS signal, 12/31 had a low to intermediate signal and 15/31 had a strong ipFDS signal. Total *B. malayi* worm burden was assessed at +41 weeks (Fig. 2A). In 3/4 mice with no ipFDS signal, no adult *B. malayi* were found. A single, motile male *B. malayi* was isolated from the other mouse in this group. In mice categorised with a low-intermediate ipFDS signal, median worm burden was 9.5 (range 3–23) and 50% of the group contained ≥10 adult *B. malayi*. In mice categorised with a high ipFDS signal, median worm burden was 19 (range 8–33) and 87% of the group had a worm burden ≥10. The difference in worm burden predicted by USG categorisation was significantly different between all sub-groups (1way ANOVA *F* = 10.82, *P* = 0.0003, Fig. 1A). After a period of between 3–6 months post-infection, the cohort of gerbils were subjected to USG and scored as the CB.17 SCID study above (Fig. 2B). Of the gerbils examined, 4/4 of the sham-infected gerbils had no detectable ipFDS. A further 5 gerbils who had received inoculates of L3 were ipFDS negative and were determined to be uninfected at necropsy. Six gerbils were ascribed a low/intermediate ipFDS signal and contained a range of 1–2 adult parasites at necropsy. The remaining gerbils scoring a strong ipFDS signal contained a median worm burden of 21 (range 12–68). Therefore, in gerbils characterized with a high ipFDS, 100% of the sub-group had a worm burden ≥ 10 adult *B. malayi*, whereas those characterised with an intermediate signal, 0% of animals in the group had a worm burden ≥10 adult *B. malayi*. In both mice and gerbils, worm masses were typically visualised underneath the liver or groin area, with number of locations found incorporated into the scoring system. The number and location of worm masses could not be accurately correlated at necropsy due to parasites being purposely dislodged by lavage and thus distributed throughout the peritoneum, to allow for easy detection by the dissector.

### Evaluation of estimated reductions in animal use for drug screening post-implementation of USG assessment

We accumulated data sets of individual adult *B. malayi* worm burdens derived in multiple preclinical experiments in our laboratory to accurately determine sample variation. Data from experimental infections of gerbils and SCID mice, as well as recovery of adults post-surgical implantation in gerbils were assessed (Table 4). From the sample means and standard deviation in adult burdens we derived minimum group sizes to assess with >75 < 90% statistical power a ≥70% or ≥90% reduction in worm burden of an effective macrofilaricidal drug. The meta-analysis demonstrated that gerbil infections had increased variation in worm burden compared to SCID mice (29.4 ± 33.1, n = 43 *versus* 15.3 ± 8.7, n = 50) and higher incidence of infection failures (20% *vs* 10%). Power calculations determined that nearly 3-fold more animals would be required if assessing ≥70% efficacy of a drug candidate in gerbils *versus* SCID mice (22 *versus* 8). As prohibitively consuming in terms of gerbil use and cost, the alternative strategy of surgically implanting 20 adult *B. malayi* into gerbil recipients from infected donors was adjudged to markedly reduce variation in resultant adult yields at assay endpoint (9.4 ± 4.4, n = 11). Whilst this meant that statistical power of assessing ≥70% efficacy was achieved with a group size of seven, when taking into account a 1:1 ratio of donors to recipients, a total gerbil use per drug test of n = 14 would be necessary. We then evaluated the potential effect of excluding light infections and/or uninfected animals via USG assessment on overall animal use. In gerbils, exclusions of uninfected and low level infections would mean that similar total numbers of animals could be used compared with surgical implantations, whilst obviating the requirement for invasive surgery, as a beneficial refinement to animal welfare (for ≥70% efficacy, 14 animals per group). For SCID mice, exclusions of uninfected and low level infections would mean that total animal use per drug group tested could be reduced by between 30–40%, depending on required efficacy level being evaluated.

**Table 4:** Meta-analysis of *B. malayi* worm burden variation, statistical power and hypothetical animal use for preclinical drug screening pre- and post-implementation of USG imaging assessment.

	Species/strain	Model	worm burden mean ± SD (sample n, expt n)	n animal/drug test (>75% < 90% power)^#^		Proportion infections excluded (%)	minimum animal use/drug test†	
				≥70% efficacy	≥90% efficacy		≥70% efficacy	≥90% efficacy
**Pre-USG assessment**	Gerbil	400x*Bm*L3 infection	29.4 ± 33.1 (43, 3)	22	14	0%	22	14
	Gerbil	20xadult*Bm* implantation	9.4 ± 4.4 (11, 2)	7	5	—	14	10
	Mouse CB.17 SCID	100x*Bm*L3 infection	15.3 ± 8.7 (50, 2)	8	6	0%	8	7
**ipFDS- excluded**	Gerbil	400x*Bm*L3 infection	37.2 ± 33.1	15	9	20%	18	11
	Mouse CB.17 SCID	100x*Bm*L3 infection	17.0 ± 7.4	5	4	10%	6	5
**ipFDS−/+/++ excluded (<10 adults)**	Gerbil	400x*Bm*L3 infection	47.4 ± 31.3	9	6	40%	14	9
	Mouse CB.17 SCID	100x*Bm*L3 infection	19.5 ± 6.0	4	3	25%	5	4

^#^Statistical power (1-ß, α = 0.05) two sample T test (Russ Lenth Piface Applet).

^†^Including donor animals (surgical implantations) or animals used but subsequently excluded due to USG criteria.

## Discussion

Intraperitoneal infections of rodents with *Brugia spp*. are convenient small animal models to test activity of candidate filaricidal compounds. However, limitations of the current screening models means large numbers of animals are required to gauge with accuracy the efficacy level of test compounds. Variation in adult parasite worm burden and the occurrence of infection failure in both gerbils and, to a lesser extent, immunodeficient mice, hampers the success of screening systems to delineate the true efficacy level of treatments. To compensate for variation in worm burden, investigators accommodate large group sizes which is costly and increases overall animal use. A second strategy to overcome parasitological variation is the surgical transfer of adult *Brugia* parasites from donor-infected animals prior to drug dosing. Whilst this strategy improves accuracy of drug efficacy evaluation, it requires an invasive procedure. A second limitation of current screening systems is that no accurate quantitative biomarker of active infection is available. Thus, both initial starting adult biomass and endpoints of drug treatment efficacy are difficult to predict. Microfilarial production can be used as a marker of fecund adult infection but is not infallible due to occurrence of single sex infections and because mature mf can persist long term after the death of adult parasites (half life~100 days). Further, sampling of mf in the peritoneum requires invasive catheter washing under anesthesia, which can also coincidently remove adult parasites, particularly male *Brugia*. Ultimately, these drawbacks mean that animals are either maintained for very long durations (up to 8 months post-drug treatment) with the concomitant risk of welfare issues arising, or multiple groups are used to sample different time points after treatment, with yet further increases in overall animal use.

Previously, USG has been used in the field of tropical medicine primarily as a diagnostic tool, and also to assess therapeutic outcomes. One example is the use of USG to detect pathology caused by certain parasites, such as assessing liver fibrosis and changes in urinary tract structure due to schistosomiasis^35^. USG has also been applied to other diseases, including visceral leishmaniasis and viral haemorrhagic fever, whereby pathological changes in different organs can be used to determine disease state and therapeutic efficacies^14^. In terms of filariasis, USG has been used in the field to detect FDS in hydrocele patients and to determine macrofilaricide and anti-morbidity drug activity^15,16,18,19,36^.

To our knowledge, there has been no literature reported on the use of USG for pre-clinical drug models of Brugian filariasis, making this research a novel expansion of the technique to determine efficacy of drug candidates and significantly reduce animal usage in this area.

We tested whether ultrasound detection of adult *Brugia* ‘filarial dance sign’ could be employed as a specific adult filarial biomarker to reduce and refine rodent use for *in vivo* filarial drug screening. After optimizing the technique for ipFDS detection in mice and gerbils, we determined, in multiple operator/operator-blinded studies that USG was 100% specific in predicting animals who were infection negative and 86% sensitive in detecting active adult *B. malayi* infection. This increased to 100% sensitivity when >5 adult motile adult parasites were present in the peritoneum. The USG technique could be mastered and transferred from one operator to another with minimal amount of training and practice and without necessary prior experience of USG. Interestingly, sensitivity of ipFDS detection was not significantly different in single sex infections when low numbers of either larger, wider female (4–5 cm × 180–230 μM) or smaller, thinner male worms (1–2 cm × 70–80 μM) were evaluated *in vivo*. Indeed, single male worms were detectable in 100% of animals tested. This highlights a remarkable sensitivity of USG to detect adult infection and illustrates sensitivity is more related to the rapidity of filarial motility rather than the size of adult worm per se. Reduction in sensitivity of USG detection reduced from 100% in animals parasitised with >5 adult worms to 81% in animals containing ≤5 worms. This probably reflects that with fewer parasite masses in fewer anatomical locations, a positive ipFDS signal is more likely to be missed over the 15 minutes of USG scanning especially if worms are situated in smaller cystic spaces surrounding solid tissues. The results indicate the technique may be able to predictively detect earlier, smaller life-cycle stages such as juvenile adult worms or even fourth-stage larvae, which may have use in determining earlier endpoints of drug- or immuno-prophylaxis type preclinical studies.

We demonstrated that USG detection of motile adult *B. malayi* could be successfully applied as an early prognostic measure of effective macrofilaricidal activity. Using flubendazole injection as a reference macrofilaricide, as little as 2.5 weeks after dosing, USG could accurately predict if a rapid-acting macrofilaricidal drug regimen was significantly efficacious in operator-blinded studies. The predictive power of the USG approach was related to both a reduced total adult worm burden but also a severely reduced motility phenotype of surviving worms following effective drug treatment. Thus, we conclude that ipFDS signal is an accurate predictor of whether a drug candidate is likely to deliver significant macrofilaricidal outcome. Implementing USG to screen animals post-treatment would therefore potentially reduce the overall length of washout post-dosing, thus mitigating against reduced welfare of protracted animal experiments and the associated risk of underpowered studies requiring reassessment.

When imaging on “B mode”, it was possible to infer a semi-quantitative worm burden based on the apparent visual mass of worm movement and also the number of discreet locations where ipFDS was detected. As well as identifying sham infections and infection failures with 100% accuracy, this also enabled USG operators to predict with significant accuracy whether an animal contained a low/moderate or high worm burden, which when compared with yield of parasites at necropsy was between 87–100% accurate at delineating animals with ≥10 adult *B. malayi*. We evaluated that if implemented prior to randomisation into drug screening experiments, with infection negative and light infection animals excluded, this semi-quantitative USG technique would reduce both intra- and inter-group variation and would thus impact on total numbers of animals required for drug screening. For gerbil-specific drug screening experiments, a major benefit of USG evaluation would be to negate the necessity of surgically implanting adult parasites from donors to recipients without increasing overall animal use. For SCID mouse experiments, animal use could be reduced as much as 40%. Further reductions in animal use over and above this would be apparent if experimental designs were altered to reflect the requirement of only a single time point for end-point analysis, after implementing longitudinal USG assessments.

In conclusion, USG is a 100% specific and highly sensitive bioimaging technique to detect adult *Brugia* filarial parasites in the peritoneum of infected rodents. The technique can be implemented with minimum training. Implementation of USG would be beneficial in terms of refining animal experiments (negating the requirement for surgery and invasive sampling) and also has the potential to reduce overall animal use by as much as 40% in the context of preclinical anti-filarial drug screening.

## Electronic supplementary material

Supplementary video S1

Supplementary video S2

Supplementary video S3
